# Intestinal *Salmonella typhimurium* Infection Leads to miR-29a Induced Caveolin 2 Regulation

**DOI:** 10.1371/journal.pone.0067300

**Published:** 2013-06-24

**Authors:** Lena Hoeke, Jutta Sharbati, Kamlesh Pawar, Andreas Keller, Ralf Einspanier, Soroush Sharbati

**Affiliations:** 1 Institute of Veterinary-Biochemistry, Freie Universität Berlin, Berlin, Germany; 2 Department of Human Genetics, Saarland University, Homburg, Saar, Germany; 3 Siemens AG, Healthcare Sector, Erlangen, Germany; University of Heidelberg Medical School, Germany

## Abstract

**Background:**

*Salmonella* are able to modulate host cell functions facilitating both uptake and resistance to cellular host defence mechanisms. While interactions between bacterial modulators and cellular proteins have been the main focus of *Salmonella* research, relatively little is known about mammalian gene regulation in response to *Salmonella* infection. A major class of mammalian gene modulators consists of microRNAs. For our study we examined interactions of microRNAs and regulated mRNAs in mammalian intestinal *Salmonella* infections using a piglet model.

**Methodology/Principal Findings:**

After performing microRNA as well as mRNA specific microarray analysis of ileal samples from *Salmonella* infected as well as control piglets, we integrated expression analysis with target prediction identifying microRNAs that mainly regulate focal adhesion as well as actin cytoskeleton pathways. Particular attention was given to miR-29a, which was involved in most interactions including Caveolin 2. RT-qPCR experiments verified up-regulation of miR-29a after infection while its predicted target Caveolin 2 was significantly down-regulated as examined by transcript and protein detection. Reporter gene assays as well as RNAi experiments confirmed Caveolin 2 to be a miR-29a target. Knock-down of Caveolin 2 in intestinal epithelial cells resulted in retarded proliferation as well as increased bacterial uptake. In addition, our experiments showed that Caveolin 2 regulates the activation of the small Rho GTPase CDC42 but apparently not RAC1 in human intestinal cells.

**Conclusions/Significance:**

Our study outlines for the first time important regulation pathways in intestinal *Salmonella* infection pointing out that focal adhesion and organisation of actin cytoskeleton are regulated by microRNAs. Functional relevance is shown by miR-29a mediated Caveolin 2 regulation, modulating the activation state of CDC42. Further analysis of examined interactions may support the discovery of novel strategies impairing the uptake of intracellular pathogens.

## Introduction

Many bacterial pathogens including *Mycobacteria*, *Listeriae*, *Shigellae* and *Salmonellae* have the ability to invade host cells and survive intracellularly. Mucosal surfaces constitute a barrier between the host organism and the environment and are often the site of entry of bacterial pathogens. The intestine in particular acts as a portal for many invasive pathogens such as *Salmonellae* that enter host cells and cause severe damage. *Salmonellae* rank among the most successful bacterial pathogens, as they are able to infect a wide range of vertebrates. *Salmonellae* associated diseases include gastroenteritis, abdominal pain, inflammatory diarrhoea and enteric fever. Among the 2500 known serotypes, only a few have limited host ranges. Many of the known non-typhoid serotypes such as *Salmonella enterica* subsp. *enterica* serovar Typhimurium (*Salmonella*) are zoonotic pathogens and have a broad host range and commonly cause human gastroenteritis. Additionally, other serotypes such as *S.* Cholerasuis or *S.* Dublin are specifically adapted to hosts such as swine or cattle, respectively, but can also infect humans [1].

After finding their way into the host gastrointestinal tract and overcoming the low gastric pH, *Salmonellae* evade host intestinal luminal defence mechanisms such as secretory IgA, antimicrobial peptides, digestive enzymes etc. by penetrating the intestinal mucous. After adherence to the apical surface of epithelium, *Salmonellae* invade non-phagocytic enterocytes of the intestinal epithelium by mediating endocytosis. Among enterocytes, M-cells in Peyer’s patches represent the main portal for host invasion. By this means non-typhoidal *Salmonellae* are able to infect epithelial cells also basolaterally and induce local intestinal inflammation. Serotypes that are capable of causing disseminated infection enter macrophages, using them as vehicles to spread through the host organism [2]. The mechanisms of *Salmonella* virulence factors that mediate invasion of intestinal epithelia are well understood. Invasion requires reversible adhesion followed by final docking via the Type III secretion system 1 (TTSS1), which is applied to inject a number of virulence factors encoded by the *Salmonella* pathogenicity island 1 (SPI-1) such as SopE [3], [4]. This effector protein acts as a guanine exchange factor for the small Rho GTPases CDC42 and RAC1, causing reorganisation of actin and inducing membrane ruffling of host cells, which lead to invasion. Moreover, SPI-2 effectors injected by TTSS2 are needed for inhibition of phagosomal maturation, preventing oxidative eradication and promoting systemic infection [4]. However, it seems that the functions of SPI-1 and 2 overlap more than previously thought [5].

Besides virulence factors, host factors such as membrane cholesterol or lipid rafts were examined to facilitate *Salmonella* invasion of host cells. It was shown that cholesterol association with TTSS components is essential for host cell membrane binding and virulence factor delivery into the host cell [6]. Interestingly, it was reported that *Salmonella* invasion in a human M-cell model is mediated by caveolae [7], which are small vesicular invaginations of the plasma membrane. They are lipid raft domains composed of cholestrol, glycosphingolipids, GPI-anchored proteins and Caveolins. The latter are members of a protein family (CAV1-3) that are predominantly found in plasma membranes but also in vesicles and cytosol. CAV1 and 2 are expressed in most tissues, while CAV2 expression seems to be depending on CAV1 presence [8]. CAV1 homo-oligomers are able to form caveolae, whereas CAV2 exists in caveolae only as hetero-oligomers together with CAV1. CAV3 is mainly expressed in muscle cells and possesses a highly similar primary structure to CAV1 [8], [9]. Caveolins have been also detected outside caveolae for example in the cytoplasm, Golgi apparatus and in focal adhesions (FAs) pointing to their role in cell signalling. For example, CAV1 is able to interact with many signalling proteins such as integrins, SRC-family kinases or small Rho GTPases [10], [11]. Several recently published articles have recognised the role of CAV1 and 2 in the interaction of host cells with bacterial pathogens such as *Pseudomonas aeruginosa*, *Neisseria gonorrhoeae*, enteropathogenic *Escherichia coli* and *Salmonella* [12]–[14]. CAV1 deficient mice infected with *Salmonella* were shown to possess significantly higher spleen and tissue burdens and to produce increased amounts of inflammatory cytokines compared with the wild type [13]. On the other hand, it was shown that phosphorylated CAV1 is able to block bacterial uptake by interaction with the guanine exchange factor VAV2 and activation of its substrate the small GTPase RhoA [12]. The role of Caveolins as signalling molecules as well as their regulation in the context of host cell invasion seems to be pathogen specific and needs further in-depth investigation.

Several recently published studies have shown that microRNAs (miRNAs) such as miR-203 or miR-199a-3p regulate both CAV1 and CAV2, respectively [15], [16]. MiRNAs belong to the class of intrinsic small non-coding RNAs, which are regarded to be key regulators of eukaryotic gene expression. They are mainly transcribed by the RNA polymerase II. Primary transcripts fold into hairpins that are processed by two cellular nucleases Drosha and Dicer. The final product is a miRNA/miRNA* duplex possessing a size of about 20 nt. The mature miRNA is single stranded and is loaded to an Argonaute family protein forming the miRNA induced silencing complex (miRISC) that interferes with respective targets. This complex recognises the target site within the mRNA and facilitates translational repression or mRNA degradation [17], [18]. Shatseva and colleagues proposed that miR-199a-3p mediates increased proliferation of virus-immortalised rat endothelial cells as well as breast cancer cells by down-regulation of CAV2, while its overexpression leads to decreased proliferation. Interestingly, they observed concomitant overexpression of CDC42 and hypothesised a possible link between miR-199a-3p, CAV2 and CDC42 potentially regulating cell proliferation [16].

While the role of miRNAs in development, cell cycling, cell fate decision or cancer was elucidated within the last 20 years, only few recent studies underline the role of miRNAs in the host response to bacterial pathogens such as *Salmonellae* or *Mycobacteria*. The let-7 family in particular has been attributed to *Salmonella* infections. A study by Schulte et al. showed that repression of the let-7 family in macrophages and epithelial cells eliminates the negative post-transcriptional control of the cytokines IL6 and IL10 [19]. Another recent study showed that infection of mice with *Listeria monocytogenes* or *Mycobacterium bovis* BCG down-regulated miR-29 expression in IFNγ-producing natural killer cells, CD4^+^ and CD8^+^ T cells. In addition, they reported that miR-29 suppresses the IFNγ-production by direct degradation of IFNγ-mRNA [20]. Also our group showed that in a mycobacterial infection model based on using human primary macrophages the major Caspases 3 and 7 are under the control of let-7e and miR-29a, respectively [21].

Along these lines, we hypothesised that mammalian intestinal *Salmonella* infection may lead to dysregulation of miRNAs governing cellular signalling cascades that could either regulate host response to *Salmonella* or be exploited by this pathogen. Here we present the first comprehensive investigation of mammalian intestinal *Salmonella* infection using a piglet model. The study included not only *Salmonella* infected and non-infected groups but also animals that were infected with *Salmonella* and co-treated with probiotics. The latter was intended to give a hint on mode of action of probiotics in the context of intestinal salmonellosis. Our study considered not only whole transcriptome analysis of protein-coding genes, but also integrated the expression of regulating miRNAs from same samples with target prediction. This approach allowed us to decipher an mRNA-miRNA-network of mammalian intestinal response to *Salmonella* infection. Besides these novel findings, we observed dysregulation of miR-29a in infected animals leading to down-regulation of CAV2. Using defined human as well as porcine cellular models we were able to show that proliferation of intestinal epithelial cells as well as *Salmonella* uptake is apparently controlled by miR-29a mediated CAV2 regulation.

## Materials and Methods

### Bacterial Strains, Cell Lines and Culture Conditions

The multiresistant *Salmonella* Typhimurium DT104 as well as the probiotic strain *Enterococcus faecium* NCIMB 10415 were cultivated as described earlier [22]. The human cervix carcinoma cell line HeLa (ATCC No. CCL-2) and the human colorectal adenocarcinoma cells HT-29 (DSMZ No.: ACC 299) were maintained in RPMI 1640 (Biochrom AG) supplemented with 10% fetal bovine serum superior (Biochrom AG) and 10 µg/ml Gentamicin (Biochrom AG) and passaged once weekly. The porcine intestinal epithelial cell line IPEC-J2 [23] was maintained in DMEM:Ham’s F12 (Biochrom AG) supplemented with 10% fetal bovine serum superior (Biochrom AG) and 10 µg/ml Gentamicin (Biochrom AG). Cultivation of cells was performed in 25 or 75 cm^2^ flasks (Greiner Bio-One GmbH) at 37°C and 5% CO_2_.

### Animal Experiments

The study was performed according to internationally recognised guidelines (EU directive 86/609/EWG) followed the German law on animal welfare (§ 8 TierSchG) and was approved by the local animal welfare committee of the Federal Ministry of Consumer Protection, Food and Agriculture (Permission no. G0037/02). Litters of crossbreed piglets (EUROC×Pietrain) were divided into three groups (C: control, S: *Salmonella* and SP: *Salmonella* and probiotics). Piglets in the control and *Salmonella* group had free access to pre-starter feed from day 15 to day 28 and starter feed from day 29 to day 56 postpartum. Piglets in group SP had free access to pre-starter and starter feed supplemented with the probiotic strain *E. faecium* NCIMB 10415 (Cylactin, DSM Nutritional Products). Pre-starter feed was supplemented with 7.5×10^6^ *E. faecium* NCIMB 10415/g feed and starter feed with 4.4×10^6^ *E. faecium* NCIMB 10415/g feed.

All piglets were weaned at day 28 postpartum. After weaning, the piglets in S and SP groups were infected with *Salmonella* Typhimurium DT104 (5–10×10^9^ CFU/piglet). Piglets were sedated by intramuscular application of 1.0 mg/kg azaperon and infected by intragastric application using a stomach tube. One piglet per litter from the control group was sacrificed on day 28, 31 and 56 postpartum (5 piglets at each time point resulting in a total of 30 animals). Two piglets per litter from the S and SP group were sacrificed on day 28 (3 h p.i.), 31 (3 d p.i.) and 56 (28 d p.i.) postpartum (10 piglets at each time point, except 8 piglets on day 56 in the SP group, resulting in a total of 78 animals). Sacrificing of piglets and intestinal tissue sample preparation from ileum were performed as described previously [24].

### RNA Extraction and Sample Preparation

For highly representative measurements, sample preparation was performed as described earlier [24]. Total RNA was isolated and quality controlled as described previously [25]. For Microarray analysis, pooled samples of each experimental group (C, S and SP) were prepared *ana partes aequalis* from individually isolated total RNA samples from ileum of piglets, which were collected at the same time point in each group, resulting in a total of 9 pooled samples. For RT-qPCR individual samples were used.

### cDNA Microarray Analysis

Gene expression analysis was performed using the whole genome microarray pigoligoarray, a swine protein annotated oligonucleotide microarray comprising 20400 70-mer oligonucleotides [26]. Microarray slides were re-hydrated three times over a 50°C water bath for 10 sec and subsequently dried at 65°C for 5 sec. UV crosslinking was performed at 180 mJ. Slides were washed in 0.1% SDS for 5 min, dipped in nuclease free water, immediately transferred to 100% ethanol, incubated for 3 minutes and dried in a centrifuge at 500 rpm for 3 min. Total RNA was converted into cDNA and fluorescently labelled using the SuperScript Plus Indirect cDNA Labelling System (Life technologies) and the Alexa Fluor®Dyes (Life technologies) as previously described [21]. Hybridisation was conducted as a two colour experiment in which cDNA of pooled samples from each treated group and time point was labelled with Alexa Fluor 647® (red emission), while a common reference was labelled with Alexa Fluor 555® (green emission) and hybridised on the same microarray. Hybridisation and washing was performed in the aHyb™ Hybridisation station (Miltenyi Biotec). First, slides were pre-hybridised at 45°C for 5 min in pre-hybridisation buffer (5×SSC, 0.1% SDS, 1% BSA). Prior to hybridisation fluorescently labelled samples were denatured for 5 min at 95°C and combined with 200 µl hybridisation buffer (37.5 mM sodium citrate, 375 mM NaCl, 20% formamide, 7% SDS and 200 µg/ml yeast RNA). Hybridisation was performed for 16 h at 50°C with a pump rate of 1 ml/min. The slides were washed in wash buffers with increased stringency as follows: first for 1 min at 50°C in wash buffer 1 (2×SSC, 0.5% SDS), then wash buffer 2 (0.5×SSC, 0.5% SDS), and wash buffer 3 (0.1%SSC, 0.2%SDS), each for 1 min at 25°C. Slides were removed from the hybridisation station, dipped several times in nuclease free water, and finally dried by brief centrifugation. Dye-specific bias effects were judged by dye swap experiments. Microarray scanning and data analysis were performed as described earlier [21] using the GenePix 4000B scanner (Molecular Devices) and the TM4 package [27]. Following microarray analysis, log 2 ratios of co-hybridised samples and a common reference were calculated to define groups of genes in multidimensional datasets and to find out the effects of ileal *Salmonella* infection on expression of protein coding genes. For this purpose, data were sorted by cluster analysis followed by determination of affected pathways. Before partitioning the mRNA microarray data by k-Means clustering (KMC), the optimal number of clusters was determined by Figure of Merit (FOM) analysis [27].

MIAME-compliant data of all performed microarrays considering the applied platforms as well as processed and raw sample data were submitted to the NCBI GEO repository [28] and accession-numbers were assigned (SuperSeries: GSE40805).

Statistical microarray data analysis as well as pathway analysis was performed as described earlier [21].

### Microfluidic miRNA Microarrays

Microfluidic microarray experiments were performed using customised Geniom biochips (febit). Customised microarrays were synthesised with the Geniom One device (febit) applying febit's standard shortmer kit for oligonucleotide synthesis as described earlier [25]. The microarray consisted of 489 previously predicted and validated porcine miRNAs. Each miRNA was synthesised in situ into 12 replicates resulting in microarrays possessing 5868 features and additional controls [25]. FOM analysis was performed as described above and revealed the optimal number of 8 clusters for KMC analysis of miRNA expression.

MIAME-compliant data of all performed microarrays considering the applied platforms as well as processed and raw sample data were submitted to the NCBI GEO repository [28] and accession-numbers were assigned (SuperSeries: GSE40805).

Statistical microarray data analysis as well as pathway analysis was performed as described earlier [21].

### mRNA and miRNA Quantification by RT-qPCR

Quantification of mRNA as well as miRNA expression by means of RT-qPCR was performed as described earlier by triplicate measurement of individual samples [21]. Normalisation of expression data was performed using the geNorm algorithm [29] calculating the geometric means of several reference genes. For normalisation of mRNA expression we used GAPDH, UBC as well as 18S rRNA all possessing stable expression. For normalisation of miRNA expression RNU6, SNORD47 as well as 5S rRNA were used as reference genes. The relative gene expression was obtained by calculating the ratios of absolute quantification values and normalisation factors provided by geNorm. The entire set of oligonucleotides used in this study is provided in the table S4. All oligonucleotides were synthesised by the Metabion AG.

### Transfection, Luciferase Reporter Assay, RNAi and Overexpression

All cells used in this study were transfected using the Nucleofector Technology (Lonza AG). Nucleofection was performed using 5×10^5^–1×10^6^ of either HeLa (Kit R) or HT-29 (Kit R) or IPEC-J2 (Kit L) using 1–2 µg reporter plasmid (pTK-Gluc derivatives, NEB GmbH), 100–200 ng normalisation plasmid (pTK-Cluc, NEB GmbH) and 100 pmol miRNA mimic according to the manufacturer’s instructions.

For generation of reporter plasmids, the identified human or porcine CAV2 target sites were obtained from hybridised oligonucleotides (Metabion AG) and were cloned in pTK-Gluc (NEB GmbH) and endotoxin-free reporter plasmids (pTKGhCAV2 and pTKGsCAV2) produced for transfection as described earlier [21]. The reporter plasmids were co-transfected with Pre-miR miRNA Precursors miR-29a (Life Technologies). The nonsense miRNA Pre-miR miRNA Precursor Negative Control #1 (Life technologies) as well as reporter plasmids harbouring the mutagenised target sites (pTKGhCAV2m and pTKGsCAV2m) were used as controls for specificity of interactions. Detection of *Gaussia* and *Cypridina* luciferase activities serving as experimental and control reporter genes were performed as described earlier using HeLa and IPEC-J2 cells for the human and porcine interaction, respectively [21].

Knock-down of human CAV2 in HT-29 was achieved using the ON-TARGETplus SMARTpool, Human CAV2 (Thermo Scientific). For knock-down of porcine CAV2 in IPEC-J2 cells, two siRNAs were custom designed (CAV2 ON-TARGET: 5′-cuauaaugaucaagggacauu-3′ and CAV2 Regular: 5′-gcaaauacgugaucuacaauu-3′), which were initially tested individually as well as a pool. Since pooled siRNAs showed enhanced activity, we employed equimolar pools for further investigations. For CAV2 knock-down, all cells were transfected with 100 pmol siRNA or nonsense controls.

For overexpression of CAV2 the human coding sequence was synthesised by optimising the codon usage and was provided by the company Geneart within the plasmid pDONRTM221. The CAV2 coding sequence was cloned in the expression plasmid pMIREX0 using the restriction sites ClaI and HindIII resulting in the generation of pMIREXhCAV2.

### Immunodetection, Rho GTPase Activation Assays and Co-IP

Immunodetection of proteins on Western Blots was performed as described earlier [24] using protein pools together with 1∶250–500 or 1∶750 dilution of the primary antibodies Rabbit-CAV2 (ab2911, purchased from Abcam) and Rabit-CDC42 (ab64533, purchased from Abcam), respectively. Detection was performed using Fusion-SL4 Spectra together with the CAPT and Bio-1D software packages (Vilber Lourmat).

For fluorescence immunocytology, cells were grown on coverslips in a 24 well plate. Cells were first washed with PBS and fixed in 4% formaldehyde for 15 min. For subsequent washing, the coverslips were rinsed briefly with PBS and incubated in PBS twice for 5 min. The blocking was performed using 1% BSA in PBS for 30 min. The primary antibody was diluted in the blocking solution (Rabbit-CAV2, 1∶100; Rabbit-CDC42 1:200) and incubation was performed for 1 h at room temperature. The washing steps were performed with PBS as described above. The secondary antibody (Goat Anti-Rabbit IgG DyLight 594, Thermo Scientific) was also diluted in blocking solution (1∶400) and coverslips were incubated for another 1 h at room temperature. After further washing steps, nuclei were stained with 200 ng/ml DAPI in PBS for 3 minutes, followed by further washing steps. Mounting was performed using 50% glycerol in PBS on glass slides, which were stored at 4°C in the dark.

Rho GTPase activation assays (G-LISA) for active GTPases CDC42 and RAC1 (G-LISA CDC42 or RAC1 Activation Assay Biochem Kit, colorimetric format, Cytoskeleton, Inc.) were performed to detect the active fraction after CAV2 RNAi as well as overexpression. HT-29 cells were transfected with miR-29a, anti-miR-29a, CAV2 siRNA, pMIREXCAV2 and nonsense as well as mock (pMIREX0) controls as described above. Protein was extracted 48 h post transfection. Non transfected cells were taken for generation of positive and negative controls. G-LISA experiments were performed according to the manufacturer’s protocol using 0.5 mg/ml or 1 mg/ml protein for CDC42 or RAC1 detection, respectively. Absorbance (OD 490) was detected using the FLUOstar Optima (BMG Labtech).

For Co-Immnoprecipitation (Co-IP), HT-29 cells (75 cm^2^) were harvested at 80% density under non-denaturing conditions by removing media and rinsing with ice-cold PBS. Then 0.75 ml of ice-cold 1× Cell Lysis Buffer (Cell Signaling Technology) was added to each culture flask. The flasks were incubated on ice for 5 minutes. Cells were scraped off and samples were sonicated (Branson Sonifier 250) on ice three times for 5 seconds each with constant duty cycle and output 1. After centrifugation for 10 minutes and 14,000 × g at 4°C supernatants were transferred to new tubes. To reduce non-specific binding to the Protein G magnetic beads (Invitrogen), cell lysate pre-clearing was performed by adding 200 µl of cell lysate to 40 µl pre-washed magnetic beads and incubation at 4°C for 2 hours. Then rabbit primary antibodies against CDC42 and CAV2 (Abcam) were added to pre-cleared cell lysates using 1∶100 dilutions and incubated with gentle rocking overnight at 4°C. The lysate and antibody (immunocomplex) solution was transferred to the tube containing washed magnetic bead pellets (40 µl) and incubated with gentle rocking for 4 h at 4°C. Magnetic beads were separated and the supernatants were stored in new tubes. The magnetic bead pellets were washed 3 times with 500 µl of 1× Cell Lysis Buffer. After washing, pellets were mixed with 30 µl 3× SDS Sample Buffer (187.5 mM Tris-HCl pH 6.8 at 25°C, 6% w/v SDS, 30% glycerol, 150 mM DTT, 0.03% w/v bromophenol blue) by vortexing. The samples were heated to 95°C for 5 minutes and centrifuged for 1 minute at 14,000×g. 18 µl of samples were loaded on 13.5% SDS-PAGE gel. Gels were blotted on PVDF membrane and proteins were detected using monoclonal mouse anti-CDC42 (Cytoskeleton) and monoclonal mouse anti-CAV2 (Invitrogen) using the dilutions 1∶250 and 1∶500, respectively. Signal was detected with peroxidase labelled anti-mouse antibody using 1∶5000 and 1∶2500 dilutions, respectively.

### Proliferation Assays

Real-time proliferation assays were performed using the xCELLigence RTCA together with E-Plate 96 (Roche). Initial experiments were performed for each investigated cell line where the optimal cell number for seeding was determined. The optimal cell number for HT-29 as well as IPEC-J2 was 2×10^4^ cells/well. Background measurement with 50 µl of medium per well was carried out. After addition of medium or cells, the plate was first incubated for 20 min at 37°C and 5% CO_2_ to ensure adaptation of pH. Following background measurement, 100 µl cell suspension (2×10^4^ cells) was seeded into each well. Medium background without cells served as a control. Measurement of electrical impedance was performed for 90 h in 10 min intervals while the medium was changed after 24 h and 48 h. Proliferation assays of HT-29 as well as IPEC-J2 were performed after miR-29a mimic and anti-miR experiments as well as specific knock-down and overexpression of CAV2 as described above. After a 20 min regeneration phase post transfection, cell suspensions were diluted to obtain 2×10^4^ cells per 100 µl and transferred to each well of an E-Plate. Each treatment was measured in six replicates. The measurement was performed for 90 h to reach the growth plateau phase, represented by maximum cell index.

### In vitro *Salmonella* Invasion Assay

HT-29 cells were seeded at a concentration of 1×10^5^ cells per well in a 24 well plate. After 24 h of Gentamicin-free culture at 37°C, cells were given fresh medium and infected with *Salmonella* for 2 h at 37°C using an MOI 5. Subsequently, cells were washed 3 times with RPMI medium and incubated with RPMI medium containing 10% FBS and 50 µg/ml Gentamicin for 1 h to kill extracellular *Salmonella*. After three successive washes the final supernatant was plated on *Salmonella/Shigella* agar to guarantee the absence of extracellular bacteria. For calculation of CFU of intracellular bacteria/infected cell, adherent cells were detached using 50 µl Accutase, resuspended in 350 µl PBS and the cell number was determined. Afterwards, infected cells were centrifuged and the pellet was lysed using 950 µl 0.1% Triton X-100/ddH2O for 20 min at room temperature. 100 µl was plated in triplicate on *Salmonella/Shigella* agar and CFU was determined after overnight cultivation.

## Results

### mRNA and miRNAs Transcriptome Analysis Integrated with Target Prediction Outlines Regulative Networks of Intestinal *Salmonella* Infection

To decipher regulative networks between miRNAs and modulated mRNAs in the context of intestinal *Salmonella* infection of a mammal, we performed mRNA as well as miRNA microarray analysis. Our approach was based on the integration of negatively correlated expression data together with target prediction. Ileal samples at 3 hours (sample designation: 3 h), 3 days (sample designation: 3 d) as well as 28 days (sample designation: 28 d) after weaning and simultaneous oral infection of piglets were analysed. Time points were chosen based on pathogenesis of *Salmonella* infection in pigs that is regarded to consist of following phases: colonisation of the intestine, invasion of enterocytes and bacterial dissemination to other organs [30]. Accordingly, Szabo et al. reported that while orally administered *Salmonella* were only partially detected at 3 h p.i., *Salmonella* were found in all pigs after 3 d p.i. After 28 d, 50% of *Salmonella* infected animals were still positive [22]. The specific time points were chosen according to the described course of infection, two time points were chosen, which represented the acute infection (3 h p.i. representing the first phase of interaction between *Salmonella* and host and 3 d p.i. representing the time point where all animals were *Salmonella* positive). Since *Salmonella* infections are in general regarded to be self-limiting, 28 d p.i. was taken as a time point representing partial relief. The study covered three individual groups of animals. The first group represented the control (C), the second was infected with *Salmonella* (S) and the third group was infected with *Salmonella* and co-treated with a probiotic strain (SP); more details on experimental design are provided in the materials and methods section.

**Figure 1:**
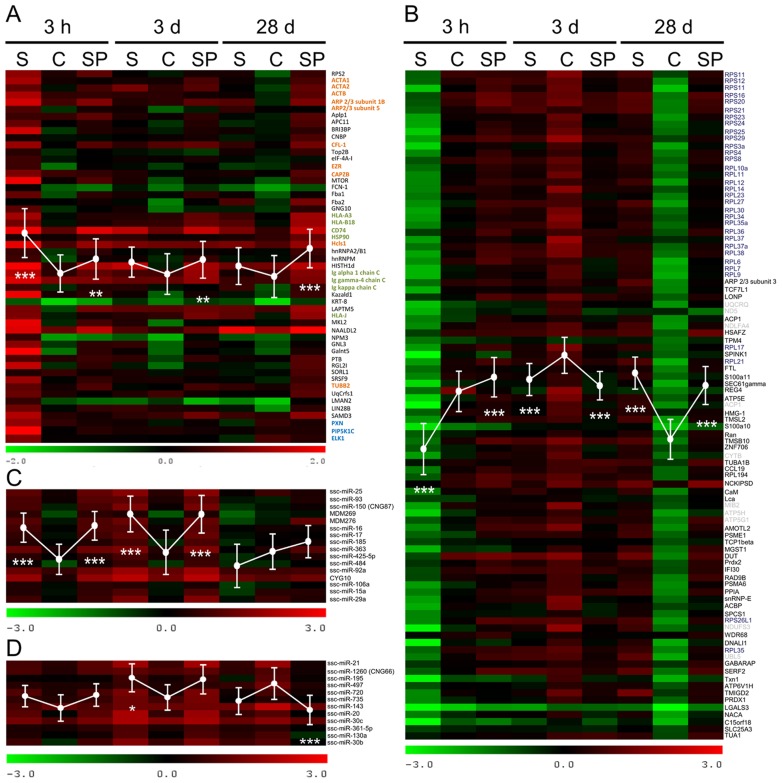
Heatmaps of exemplified mRNA and miRNA expression data clustered after microarray analysis. Columns (A–D) represent temporal expression of ileal samples collected from *Salmonella* infected (S), *Salmonella* infected and co-treated with probiotics (SP) and non-infected controls (C) at 3 h, 3 d and 28 d p.i. Colours represent log 2 ratios of the respective samples versus the common reference according to the scales shown below. Samples represent a pool of at least five infection experiments. An averaged trace of the expression profile (± SD) is integrated as a white graph. Gene clusters showing significantly differential expressions in corresponding time points p.i. were identified by the analysis of variance and Kruskal-Wallis and Dunn's post test (*: P<0.05; **: P<0.01; ***: P<0.001). Panel A and B exemplify two clusters of identified protein coding genes. Colours indicate genes involved in similar pathways (orange: pathogenic *E. coli* infections and regulation of actin cytoskeleton; green: immune response related pathways; blue: focal adhesion; gray: oxidative phosphorylation; purple: ribosome). Panel C and D illustrate miRNAs being induced after infection or showed balanced expression, respectively.

This analysis resulted in definition of 12 protein coding gene clusters composed of 835 genes that were expressed in all samples post infection (p.i.). The log 2 ratios varied between −4.5 and 3.5 (0.04 to 11 fold). KMC analysis resulted in clusters comprising between 35 and 114 protein coding genes according to respective expression patterns. Gene lists of all determined clusters including the log 2 ratios are provided in table S1. In general, distinct dysregulation was observed in group S during acute infection (3 h p.i.) compared with group C, while there were no striking effects of probiotics treatment. In figure 1 two heat maps of protein coding gene clusters are presented showing groups of up-regulated (figure 1 A) as well as down-regulated genes (figure 1 B) at 3 h after *Salmonella* infection. Gene clusters showing significant differential expression in corresponding time points p.i. were identified by analysis of variance (Kruskal-Wallis and Dunn's post test). At 3 h p.i. the cluster shown in figure 1 A was on average significantly 2.4 fold up-regulated in group S (P<0.0001) while group SP showed averaged 1.4 fold increased expression (P<0.001) compared with C. In later time points p.i. only SP possessed significantly increased expression compared with the respective controls (P<0.001). To find out if the clustered genes are accumulated in specific signalling pathways, we performed a DAVID analysis [31] using the human annotation as background. Genes that clustered in the heat map shown in figure 1 A were involved in following KEGG pathways: pathogenic *E. coli* infections (P<0.0001), regulation of actin cytoskeleton (P<0.001), antigen processing and presentation (P<0.01), Fc gamma mediated phagocytosis (P<0.01) and focal adhesion (FA) (P<0.07). Genes that were categorised in each of mentioned pathways are summarised in (table S1). Interestingly, while *Salmonella* infection at 3 h p.i. caused on average 0.4 fold down-regulation of protein coding genes that clustered in figure 1 B (P<0.0001), there was a mean 1.4 fold up-regulation in SP (P<0.0001) compared with C. 28 d p.i. both groups S and SP possessed increased expression of respective genes (3.8 and 3 fold, P<0.0001) compared with the control. DAVID analysis revealed members of the second heat map (figure 1 B) to be accumulated in KEGG categories ribosome (P<0.0001) and oxidative phosphorylation (P<0.0001). All determined clusters including genes involved in identified pathways are summarised in table S1.

After miRNA specific microarray analysis, we identified a total of 131 porcine miRNAs that were expressed in all samples and possessed log 2 ratios between −3.0 and 3.1 (0.13 to 8.57 fold). The clusters included between 9 and 25 miRNAs. As exemplified in figure 1 C, a miRNA cluster was identified showing on average 2 fold increased expression in both groups S and SP compared with the control at time points 3 h and 3 d p.i. (P<0.0001). 28 d p.i. there was no significant difference between the investigated groups. On the other hand, we also determined ileal miRNA clusters that showed overall balanced expression among all groups and time points (figure 1 D). A detailed summary of the identified clusters including log 2 ratios and ANOVA statistics is given in table S2.

For a global target analysis followed by determination of potentially affected pathways, we selected 49 miRNAs from our data that were conserved between humans and pigs and possessed mean log 2 ratios ≤ −0.5 and ≥0.5 between C and S as well SP only in early time points (3 h and 3 d p.i.). The dataset was first subjected to the intersecting miRanda and Target Scan analysis implemented in the MAGIA query [32] (not considering the expression data). This resulted in selection of 24 miRNAs with 695 respective targets. Predicted targets were then subjected to DAVID pathway analysis using the human annotation. Interestingly, most predicted targets were enriched in KEGG pathways already identified by mRNA microarray experiments described above. While 29 predicted targets were enriched in the KEGG category pathways in cancer and 20 in MAPK signalling, 19 predicted targets were accumulated in FA (P<0.0001) and regulation of actin cytoskeleton (P<0.0003), respectively.

**Figure 2:**
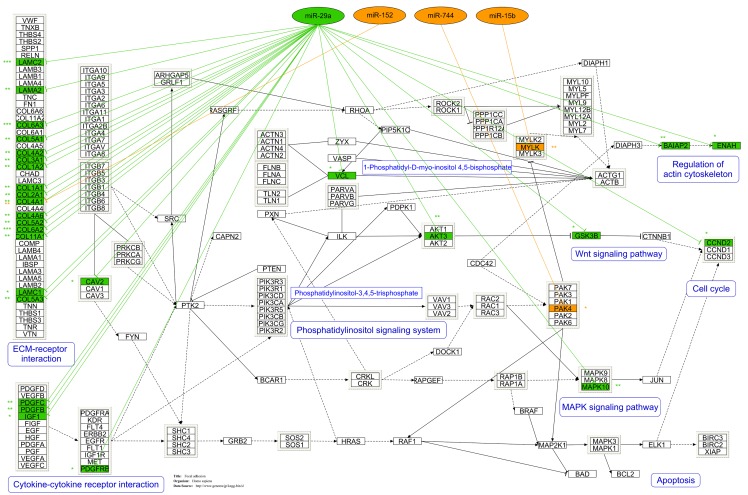
Predicted regulation of focal adhesion and actin cytoskeleton by microRNAs during intestinal *Salmonella* infection. The figure shows the first regulative concept of focal adhesion and actin cytoskeleton pathways in intestinal *Salmonella* infection of mammals. Interactions between miRNAs and mRNAs are based on the microarray study described above and were proved by RNAhybrid analysis as shown by calculated P values (*: P<0.05; **: P<0.01; ***: P<0.001).

Apart from pathway analysis, we integrated both datasets (matched mRNA as well as miRNA expression data) together with miRNA target prediction to draft regulative networks of intestinal *Salmonella* infection. This was facilitated using the analysis tool of MAGIA as described previously [21]. Because MAGIA uses human miRNA annotation, we first selected miRNAs from our data that were conserved between humans and pigs and considered only this subset for further analysis. This analysis revealed a total number of 842 potential interactions. For reduction of data and to concentrate on important regulative interactions, we only considered miRNAs and respective target genes showing anti-correlated expression that possessed correlation coefficients (Pearson r) below –0.5 (table S3). The resulting 248 interactions between 44 miRNAs and 89 protein coding genes were visualised as a network (figure S1) using Cytoscape [33]. Among the identified targets, a maximum number of four genes were accumulated in KEGG pathways. COL4A1, IGF1, MYLK and PAK4 turned out to be involved in FA while ENAH, PAK4 and MYLK were part of the pathway regulation of actin cytoskeleton. The network also included miR-29a that possessed an averaged Pearson r of −0.73 with seven identified targets including ENAH (figure S1). This particular miRNA was reported in the context of host response to bacterial infections [20], [21] as well as in regulation of actin cytoskeleton [34] and FA proteins such as CDC42 [35], which is an important factor for cellular *Salmonella* infection. To determine the role of identified interactions on these two particular pathways, we selected all first network neighbours (miRNAs) of COL4A1, ENAH, IGF1, MYLK and PAK4 and added potential Target Scan predicted miR-29a targets that were defined to be involved in both pathways (FA and regulation of actin cytoskeleton) but were not considered by our data. The resulting dataset consisted of 45 targets being involved in FA as well as regulation of actin cytoskeleton and potentially controlled by 26 of identified miRNAs (data not shown). For the identification of most probable interactions, we evaluated the entire set of miRNA-mRNA hybridisations employing individual RNAhybrid calculations [36] and discarding non-significant interactions. The RNAhybrid algorithm is able to calculate a p-value for each predicted duplex between a miRNA and a target sequence with a certain minimum free energy based on the probability that such a minimum free energy or a better one occurs by chance [37]. Evaluated interactions were visualised using PathVisio 2 [38] by considering the KEGG pathway FA as a basis. Thereby, we were able to outline the first regulation pathway between miRNAs and mRNAs in intestinal *Salmonella* infection of mammals (figure 2). We identified a total of 36 significant interactions with FA members, while 33 were assigned to miR-29a. Many potential targets of this particular miRNA clustered in extra cellular matrix (ECM) receptor interaction; miR-29a showed e.g. a calculated significant interaction with COL4A1 (mfe: −24.1 kcal/mol; P<0.002), while this target was also predicted to be co-targeted by miR-152 (mfe: −22.8 kcal/mol; P<0.01). But miR-29a was also predicted to significantly interact with major FA members such as Vinculin (mfe = -20 kcal/mol; P<0.05) and Caveolin 2 (mfe = –19.4 kcal/mol; P<0.05). However, Caveolin 1 analysis revealed no interaction with the miR-29 family (figure 2). Since most interactions were assigned to miR-29a, we speculated about its role as a potential master regulator of FA and reorganisation of actin cytoskeleton in *Salmonella* infection.

### *Salmonella* Infection Leads to Ileal miR-29a Up-regulation and Decreased CAV2 Expression

**Figure 3:**
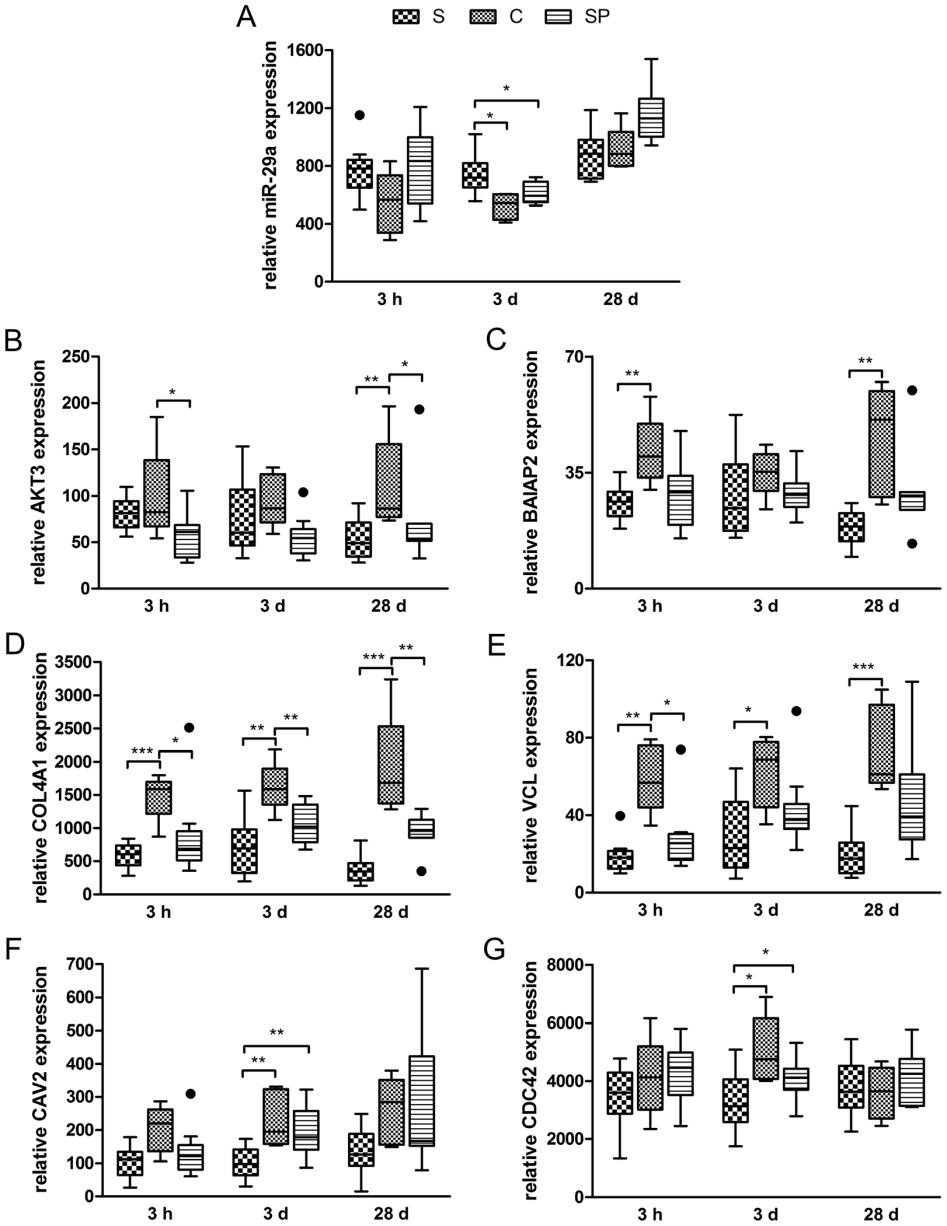
Expression analysis of miR-29a and selected targets by means of RT-qPCR. The Tukey box plots show relative mean expression of individual samples each measured in triplicates while outliers are shown as black dots (S: *Salmonella* infected; C: non-infected control; SP: *Salmonella* infected and co-treated with probiotics). Asterisks indicate statistical significance according to Mann-Whitney U test (*: P<0.05; **: P<0.01; ***: P<0.001). Panels A, B, C, D, E, F and G show the expression of miR-29a, AKT3, BAIAP2, COL4A1, VCL, CAV2 and CDC42 at 3 h, 3 d and 28 d p.i., respectively.

It is known that miRNAs cause not only translational repression but also mRNA degradation. For validation of outlined interactions (figure 2) and to legitimate calculated miR-29a-target interactions and to prove biological significance, we examined the expression of miR-29a, AKT3, BAIAP2, COL4A1, VCL, CAV2 and CDC42 over the course of infection by means of RT-qPCRs. During early infection (3 h p.i.) both groups S and SP showed averaged 1.5 fold up-regulation of miR-29a compared with C. After three days, *Salmonella* infection of piglets caused a significant 1.4 fold increased expression of miR-29a only in group S compared with C (P<0.05, Mann-Whitney U test). 28 d p.i. no significant differences were observed among the groups (figure 3 A). Furthermore, our RT-qPCR data showed that both predicted miR-29a targets AKT3 and BAIAP2 possessed an anti-correlated trend during early infection (figure 3 B and C). Another two predicted targets, the type IV alpha collagen COL4A1 and the cytoskeletal FA molecule VCL were clearly down-regulated in both *Salmonella* infected groups compared with non-infected controls (figure 3 D and E). Concomitantly decreased mRNA levels of predicted targets together with increased miR-29a expression upon *Salmonella* infection pointed out the robustness of predicted regulative pathways shown in figure 2.

In particular, we became interested in the potential regulation of a major FA factor Caveolin 2 (CAV2) by miR-29a during *Salmonella* infection. Caveolins have been identified to be involved in the pathogenesis of several bacterial pathogens and seem to play important roles in host cell interaction [12]. Both CAV1 and CAV2 were recently associated with uptake of bacterial pathogens. Therefore and to continue evaluation of the outlined regulation pathways (figure 2), we focused our proceeding work on the CAV2-miR-29a interaction. To corroborate our hypothesis that miR-29a regulates the expression of CAV2, we performed RT-qPCRs detecting the ileal expression of CAV2 upon *Salmonella* infection. This analysis showed averaged 0.52 and 0.66 fold down-regulation of CAV2 in the groups S and SP compared with C at 3 h p.i., while there was no significant difference between medians (figure 3 F). However, CAV2 turned out to be 0.52 fold significantly down-regulated on day 3 p.i. only in *Salmonella* infected animals compared with C (P<0.01, Mann-Whitney U test). According to the miR-29a RT-qPCR experiments described above, there was no significant dysregulation of CAV2 among groups on day 28 p.i. (figure 3 F). The expressions of miR-29a and CAV2 in the group S on day 3 p.i. were clearly anti-correlated possessing a Pearson r of −0.8 (P<0.05, two-tailed). Moreover, the Rho GTPase CDC42 that is known to be important for *Salmonella* uptake was already reported to be a target of miR-29a in HeLa cells [35]. Therefore we also proved the expression of CDC42 in same samples. As shown in figure 3 G, infected piglets exhibited a 0.65 fold significant decreased expression of CDC42 on 3 d p.i. (P<0.05, Mann-Whitney U test) compared with C, while there was no significant difference between SP and C. Since CAV1 has been reported to be a CDC42 guanine dissociation inhibitor (GDI) [39] and although our analysis revealed no interaction between the paralogue CAV1 and miR-29a, we analysed CAV1 expression in same samples and time points. CAV1 was expressed in porcine ileum, however, there was no consistent and significant regulation of CAV1 compared with CAV2 (values represent relative mean expression ± SD; C 3 h p.i: 241.5±71.5; S 3 h p.i.: 165.4±108.8; C 3 d p.i.: 283.4±162; S 3 d p.i.: 235.2±170.1).

**Figure 4:**
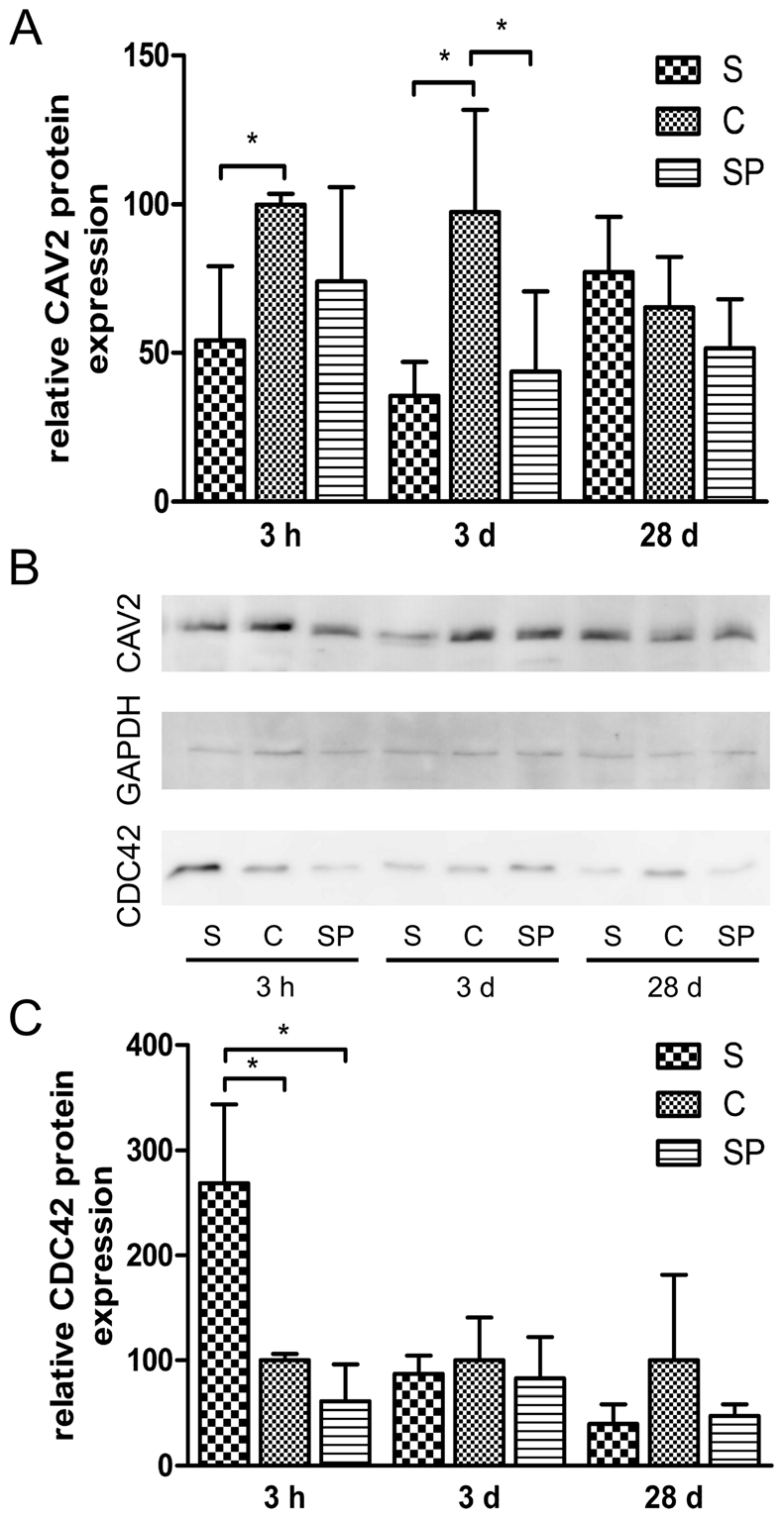
Western Blot analysis of CAV2 and CDC42 after intestinal *Salmonella* infection. Panels A–C show immuno detection of CAV2, CDC42 and GAPDH on Western Blots at 3 h, 3 d and 28 d p.i. (S: *Salmonella* infected; C: non-infected control; SP: *Salmonella* infected and co-treated with probiotics). Panel B exemplifies detection of respective proteins in pooled ileal samples from individually infected animals while panels A and C show relative mean protein expression (CAV2:GAPDH and CDC42:GAPDH) calculated from quadruplicate Western Blots and error bars show the standard deviation (*: P<0.05, Mann-Whitney U test).

Because the RT-qPCR results underlined our observations described above, we further proved the respective CAV2 as well as CDC42 protein expressions in *Salmonella* infected animals compared with C and SP. As shown in figure 4 A and B, normalised quantification (CAV2:GAPDH) of quadruplicated Western Blot analyses revealed an approximately 0.54 fold significant decrease of CAV2 protein in the group S at 3 h p.i. compared with the control (P<0.05, Mann-Whitney U test). Interestingly, CAV2 protein showed a more pronounced reduction on 3 d p.i. in both groups S and SP. While there was a 0.36 fold decrease in group S, the probiotic treated group SP exhibited 0.44 fold lower CAV2 protein (P<0.05, Mann-Whitney U test). There were no significant differences among the groups on day 28. We also quantified the normalised CDC42 protein level (CDC42:GAPDH) by quadruplicated Western Blot analyses. While there was 2.7 fold up-regulation of CDC42 protein only at 3 h p.i. in group S compared with C (P<0.05, Mann-Whitney U test) no significant differences were noticed at other time points and samples (figure 4 B and C).

Taken together, the findings from our miRNA, mRNA as well as protein expression studies supported the predicted regulative pathway analysis (figure 2) pointing to CAV2 as a bona fide miR-29a target.

### CAV2 is a miR-29a Target

**Figure 5:**
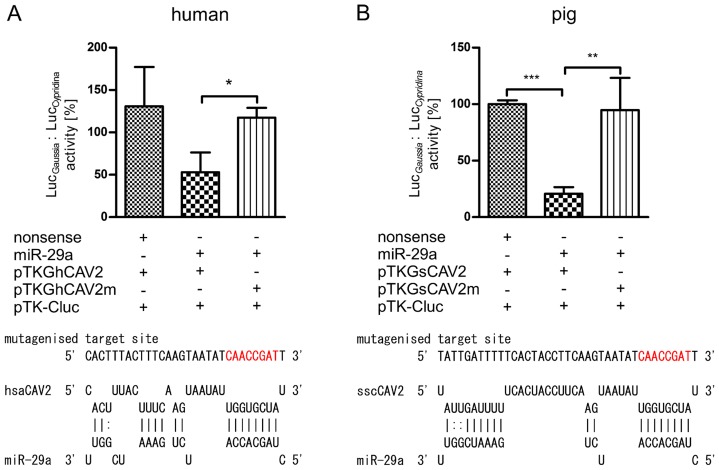
Analysis of CAV2-miR-29a interaction in humans and pigs by means of reporter gene assays. The interaction between miR-29a and human as well as porcine CAV2 was verified by means of reporter gene assays using HeLa and IPEC-J2 cells, respectively. Identified target sites between miR-29a and human (panel A) as well as porcine CAV2 (panel B) were analysed using RNAhybrid. Relative luciferase activity (Luc *_Gaussia_*: Luc *_Cypridina_*) was determined respective to nonsense miRNA mimics as well as mutagenised seeds (red letters) serving as controls. The columns show means of normalised luciferase activity each measured in triplicates while error bars show the standard deviation. Asterisks indicate statistical significance between samples (*: P<0.05; **: P<0.01; ***: P<0.001, paired t test).

As mentioned above, miR-29a is conserved between humans and pigs. For evaluating the interaction between miR-29a and CAV2 in both species, we screened the 3′ UTR of porcine as well as human CAV2 for potential miR-29a target sites. Using RNAhybrid, we were able to identify respective target sites possessing conserved seed sequences both in the human and porcine 3′ UTR. Determined target sites possessed 83.3% sequence identity between both species. Calculated minimal free energies for the human and porcine interaction with miR-29a amounted to −19.5 and −21.4 kcal/mol, respectively (figure 5). Each target site and respective controls possessing mutagenised seed regions (figure 5) were fused to a *Gaussia* luciferase and normalised reporter assays were performed by co-transfection with miR-29a mimics or nonsense miRNAs together with a plasmid encoding the *Cypridina* luciferase serving for normalisation (Luc *_Gaussia_*: Luc *_Cypridina_*). After co-transfection of miR-29a mimic together with either human or porcine reporters, a significant 0.53 fold reduction in activity was measured for the human target site (P<0.05, paired t test) while the porcine target site caused a significant 0.21 reduction in luciferase activity (P<0.01, paired t test) compared with mutagenised controls. Target sites co-transfected with nonsense miRNA showed unaffected activity (figure 5). This experiment proved the interaction between human as well as porcine CAV2 target sites and miR-29a to be specific. Furthermore, it showed that miR-29a regulation of CAV2 is conserved among both species.

**Figure 6:**
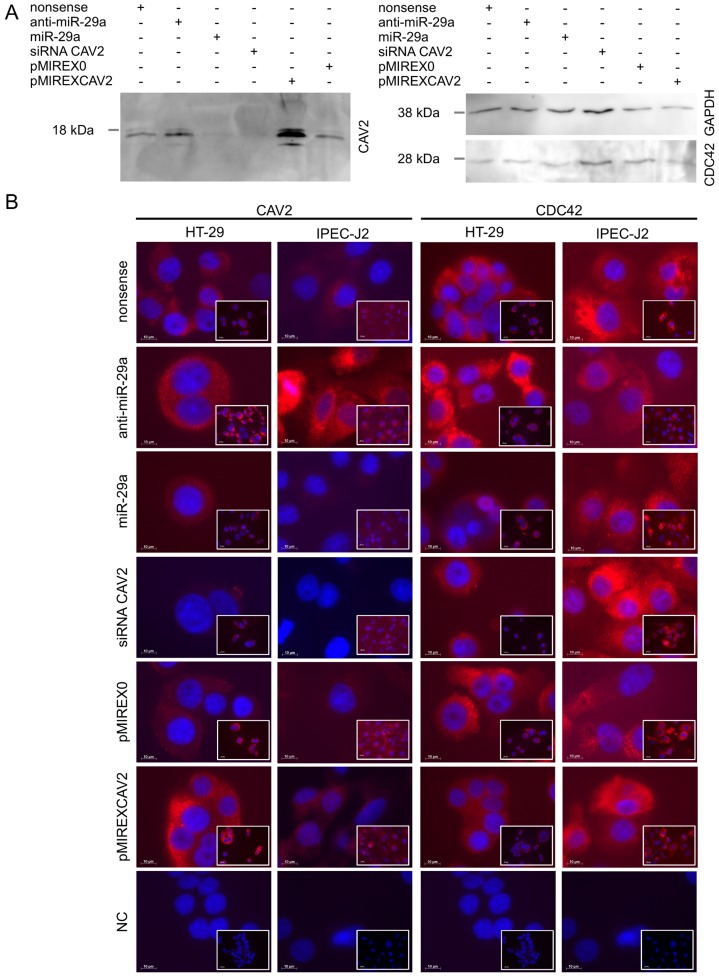
Immuno detection of CAV2 and CDC42 after RNAi and overexpression experiments in HT-29 and IPEC-J2 intestinal cells. Panel A exemplifies the expression of CAV2 as well as CDC42 after RNAi (nonsense controls, anti-miR-29a, miR-29a and siRNA CAV2) and transient overexpression of CAV2 (pMIREX0 as a mock control and pMIREXCAV2) in HT-29 cells. Panel B shows immunocyto detection of CAV2 and CDC42 after transfection (according to panel A) in intestinal cell lines of both investigated species human (HT-29) and pig (IPEC-J2). Scales are indicated by white bars (10 µm). Overview images are included in the bottom right corner.

We proved the luciferase results by knock-down of intrinsic CAV2 expression in the human intestinal epithelial cell line HT-29 using miR-29a mimics and anti-miRs as well as nonsense and CAV2 specific siRNA as controls. As shown in figure 6 A, CAV2 specific Western Blots proved the modulation of intrinsic CAV2 levels by miR-29a. While more protein was detected after anti-miR transfection, CAV2 levels were clearly diminished by miR-29a as well as CAV2 specific siRNA (figure 6 A). To find out if there is a relation between CAV2 and CDC42 protein levels we also performed CDC42 specific Western Blots by extending our investigations using HT-29 transiently overexpressing CAV2. As shown in figure 6 A, CDC42 levels in intestinal cells did neither differ by manipulation of CAV2 expression nor by miR-29a mimic and anti-miR. We extended our RNAi as well as overexpression experiments by using the porcine intestinal epithelial cell line IPEC-J2 and performing immunocyto detection of CAV2 as well as CDC42. As shown in figure 6 B, miR-29a down-regulated cellular CAV2 in HT-29 as well as IPEC-J2 while the anti-miR reversed the effect more clearly. CAV2 specific siRNA showed similar knock-down in both cell lines compared with miR-29a, while overexpression of CAV2 resulted in clearly increased fluorescence signal. To further examine potential effects of CAV2 on CDC42 protein levels, we additionally performed CDC42 specific immuno detection in similarly transfected cells. However, cellular CDC42 levels remained unaffected after both siRNA directed specific knock-down of CAV2 as well as overexpression. Additionally, miR-29a or anti-miR transfection of intestinal cell lines did not affect cellular CDC42 levels markedly when compared with mock transfected controls (figure 6 B).

### CAV2 Knock-down Leads to Retarded Proliferation of Intestinal Epithelial Cells and Increased Invasion of *Salmonella*

**Figure 7:**
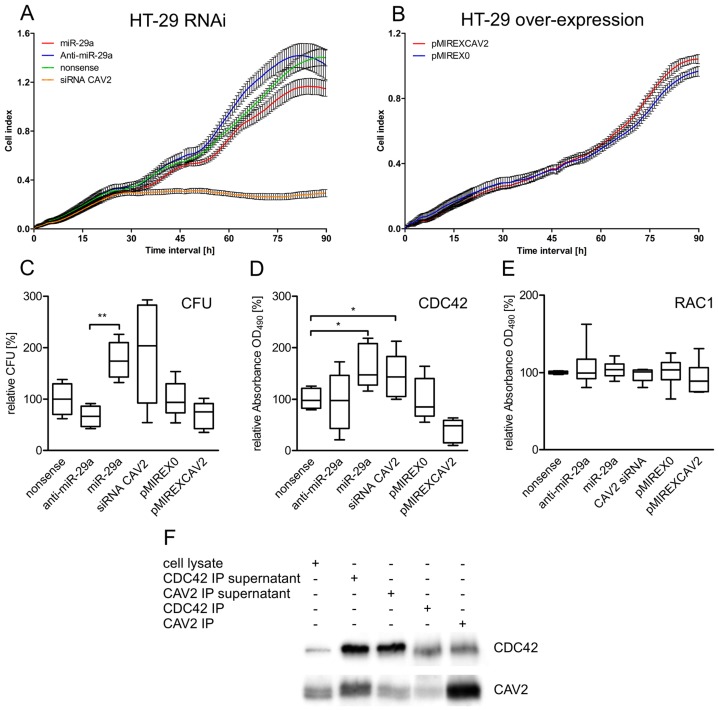
Proliferation, *Salmonella* invasion and Rho GTPase activation assays after RNAi and overexpression experiments. Panels A–B show real-time proliferation assays using the xCELLigence RTCA system (Roche) after RNAi and overexpression of CAV2 in human HT-29 intestinal cell line. Graphs show means of individually treated samples while bars indicate the standard deviation of six individually measured replicates. Panel C shows *Salmonella* invasion assays using HT-29 after RNAi and overexpression of CAV2. Individually transfected cells were infected with *Salmonella* at 48 h post transfection (five infection experiments per transfection regime, each plated in triplicate) to ensure proper CAV2 knock-down. The uptake per cell was determined as relative CFU (i.e. CFU per viable HT-29) 2 h post infection represented by box plots. Panel D and E show activation (G-LISA) assays for the small Rho GTPases CDC42 and RAC1 by detecting the active (GTP bound) fraction of cellular CDC42 or RAC1 in HT-29. Box plots indicate five replicates after RNAi and overexpression of CAV2. (*: P<0.05; **: P<0.01, paired t test). Panel F shows Co-IP experiments, where either CAV2 or CDC42 were first immunoprecipitated followed by individual detection of both proteins by means of Western Blots. CAV2-CDC42 protein interaction is pointed out by mutual co-precipitation.

Recent studies reported diverse effects of CAV2 on cell proliferation [16], [40], [41]. To learn more about potential effects of CAV2 on proliferation of intestinal epithelial cells, we down-regulated CAV2 in HT-29 and IPEC-J2 intestinal cell lines by means of CAV2 specific siRNAs as well as using miR-29a possessing rather pleiotropic effects. As additional controls we also considered same cell lines transfected with anti-miR-29a as well as transiently overexpressing CAV2. The proliferation of transfected cell lines was dynamically monitored and cell growth curves were automatically recorded on the xCELLigence System (Roche) in real time. siRNA directed specific knock-down of CAV2 led to dramatically retarded proliferation of HT-29 (figure 7 A). Cells transfected with CAV2 siRNA stopped proliferation at 30 h post transfection while no increase was observed until the end of the experiment (90 h). As indicated by the measured cell index shown in figure 7 A, HT-29 transfected with miR-29a mimic showed significantly diminished proliferation rates after 50 h compared with the control cells that were transfected with nonsense miRNA. Interestingly, inhibition of miR-29a by the antagonistic RNA anti-miR-29a led to significantly increased proliferation rates at 60 h post transfection. At the end of the experiment (90 h) siRNA as well as miR-29a transfected HT-29 possessed clearly lower cell indexes compared with controls. These pro-proliferative effects of CAV2 were confirmed by forced overexpression of CAV2 in transiently transfected HT-29. At 70 h post transfection cells overexpressing CAV2 showed clearly increased cell indexes indicating enhanced proliferation rates compared with the mock transfected HT-29 (figure 7 B). As shown in figure S2, 50 h after transfection miR-29a caused clearly decreased proliferation rates of the porcine intestinal cell line IPEC-J2 compared with nonsense transfected as well as anti-miR treated controls. Here siRNA mediated specific CAV2 knock-down as well as forced CAV2 overexpression tended to have similar effects as shown in HT-29 (figure S2). We also investigated migration characteristics of cells after knock-down or overexpression of CAV2 but there were no obvious effects (data not shown).

We also analysed potential effects of miR-29a as well as siRNA directed CAV2 modulation on *Salmonella* invasion of intestinal epithelia using HT-29 as an in vitro infection model. For this purpose, we performed RNAi experiments using CAV2 siRNA, miR-29a, anti-miR-29a and nonsense miRNA. Additional infection experiments were performed with HT-29 transiently overexpressing CAV2. Since our experiments revealed that proliferation rates differed significantly between miR-29a mimics and inhibitors after 50 h, HT-29 were infected with *Salmonella* at 48 h post transfection ensuring proper CAV2 knock-down as well as identical cell numbers among differently treated groups. The uptake per cell was determined as relative CFU (i.e. CFU per viable HT-29) right after infection. miR-29a and siRNA mediated down-regulation of CAV2 in intestinal epithelial cells led to increased uptake of *Salmonella*. While miR-29a transfection caused 1.76 fold (±0.36) increased uptake of bacteria, we determined 0.66 fold (±0.2) decrease and 1.91 (±0.99) increase of intracellular *Salmonella* after anti-miR-29a and CAV2 siRNA compared with nonsense transfected controls, respectively (figure 7 C). On the other hand, forced CAV2 overexpression caused a 0.7 fold (±0.25) reduction of the bacterial load compared with mock transfected HT-29 (figure 7 C), pointing to a direct role of CAV2 on pathogen uptake in epithelial cells.

### CAV2 Regulates the Activation State of the Small Rho GTPase CDC42

As mentioned above, CAV1 has been already identified as a CDC42 GDI in human pancreatic beta cells [39]. The authors identified a 10-amino acid region (amino acids: 92 to 101) of high conservation (FTVTKYWFYR) between CAV1 and known mammalian GDIs, which proved to be responsible for CDC42-GDP binding [39]. We therefore searched for such motifs in human as well as porcine CAV2. We were able to define a similar region in both species (amino acids: 77 to 86) possessing the following primary sequence: FEISKYV [M, human] [I, pig] YK. The porcine sequence differed only at position 84, having an isoleucine instead of methionine. This motif was 40% identical with the CAV1 region while 67% of differing amino acids were favoured or neutral displacements according to Betts and Russell [42].

Based on this observation and the knowledge that CAV2 builds hetero-dimers with CAV1 we hypothesised that CAV2 exerts similar or synergetic GDI functions as already published for CAV1. Since our RNAi experiments revealed no changes in cellular levels of CDC42, we investigated the active fraction of CDC42 after RNAi as well as CAV2 overexpression in HT-29. As shown in figure 7 D, GTP bound active CDC42 fractions increased significantly in comparison with nonsense transfected controls after miR-29a (1.63±0.38 fold) as well as CAV2 siRNA (1.44±0.39 fold) mediated down-regulation of CAV2 in HT-29 cells (P<0.05, paired t-test). The opposite effect was observed after overexpression of CAV2 or transfection with anti-miR-29a. Transient overexpression of CAV2 reduced the fraction of active CDC42 bound to GTP 0.37 fold (±0.23) and the antagonistic miRNA resulted in 0.75 fold (±0.37) active CDC42 compared with mock transfected controls (figure 7 D) while total CDC42 fractions remained unchanged after transfections (figure 6 A). These results suggest that CAV2 plays a direct role in the activation cascade of CDC42 by exerting an inhibitory effect. Since another small Rho GTPase RAC1 is known to be important for *Salmonella* induced reorganisation of actin cytoskeleton, we performed RAC1 specific activation assays after similar RNAi and CAV2 overexpression studies. However, there was no obvious effect on the activation state of RAC1 (figure 7 E). Further analysis of interactions between CAV2 and CDC42 in HT-29 by means of Co-IP experiments revealed that CDC42 is co-precipitated with CAV2 and vice versa, proving the presence of cellular aggregates, in which both proteins seem to be involved (figure 7 F).

## Discussion

Environmental interaction between species is one of the main aspects promoting evolution and directing proper development of organisms. In mammals, the interaction with commensal and environmental microbiota plays a key role in the development of the immune system. To counter this, pathogens have evolved mechanisms to destroy cellular integrity, exploiting the infected host and threatening its health. In this context, the mammalian intestinal epithelium faces an enormous challenge to preserve barrier integrity, which is achieved by gut epithelial renewal based on interplay between proliferation and migration. While proliferative renewal takes place in the crypts, epithelial cells differentiate and migrate along the villus axis and are removed by apoptosis at the tips [43]. The major apparatus regulating cell migration is regarded to be FA, a highly dynamic structure characterised by a continuous exchange of proteins such as FA kinase (FAK), Vinculin (VCL) or Paxillin (PXN) [10]. Interestingly, our microarray study showed that FA proteins such as Paxillin are up-regulated immediately after *Salmonella* infection. FAK was recently reported to promote intestinal wound healing by regulating intestinal epithelial proliferation and survival via up-regulation of Cyclin D1 [44]. Another FA protein, tyrosine phosphorylated CAV1 (pY14CAV1) was reported to enable migration and cell signalling by stabilising FAK exchange that in turn results in FA disassembly and turnover [45]. In addition, pY19CAV2 was shown to co-localise with activated FAK at FAs, suggesting convergent signalling functions [46]. Interestingly, we were able to show that CAV2 knock-down or forced expression directly reduces or enhances proliferation of human as well as porcine intestinal epithelial cells in vitro, respectively. Recently, it was shown that CAV2 has pleiotropic effects on proliferation of tumour cells. The authors showed that CAV2 expression in either neuroblastoma or hepatocellular carcinoma cells, as well as its knock-down in glioma cells, leads to reduced proliferation [40]. These observations point out that although Caveolins are a well-known FA protein family, their functions as signalling molecules remain poorly understood [10] and their signalling cascades seem to exert cell lineage dependent effects. miR-29a-CAV2 mediated decrease of intestinal epithelial proliferation, as shown by our in vitro studies, may slow-down gut epithelial renewal causing delayed wound-healing of infected tissue and exert beneficial effects for bacterial pathogens. Further in vivo studies are needed to prove this hypothesis.

FAs are defined as bidirectional mechanical biosensors enabling environmental sensing and integrating intracellular and extracellular stimuli [10]. While enterocytes communicate with the *lamina propria mucosae* basolaterally, they have to sense the environment on the apical site, suggesting that FA-like structures serve as a potential tool for this purpose. Interestingly, FA-like structures between enterocytes and *Salmonella* were shown to be important for uptake at the apical pole (free from integrins) of intestinal cells. It was shown that FAK as well as p130CAS are necessary for bacterial uptake via formation of FA-like structures [5]. Our integrated analysis of miRNA and mRNA expression in intestinal *Salmonella* infection pointed out that FA members such as VCL and CAV2 but also ECM proteins that are involved in FA formation are controlled by miRNAs, particularly by miR-29a. Accordingly, our RT-qPCR data showed decreased COL4A1 as well as VCL expression upon *Salmonella* infection. In a recent study, *Salmonella* faecal shedding or tissue colonization in pigs was related to single nucleotide polymorphisms in VCL [47]. Furthermore, our analysis revealed that factors such as ENAH and BAIAP2 that are associated with the regulation of the actin cytoskeleton seem to be under the control of miR-29a. Our results showed significantly decreased BAIAP2 mRNA levels in the *Salmonella* infected group. BAIAP2 is known to be associated with small Rho GTPases and is involved in lamellipodia and filopodia formation. It was shown that *Salmonella* promotes the formation of a BAIAP2/WAVE2 complex that leads to invasion of polarised cells [48]. On a different note, AKT signalling was shown to play a protective role in *Salmonella* infection [49]. Interestingly, our data suggest that AKT3 is regulated by miR-29a upon infection. Taken together, these results point out the robustness of predicted interactions. Hence, the determined regulative networks provide a thorough basis for deciphering how cellular signalling cascades are regulated upon *Salmonella* interaction.

Interestingly, miR-29a was reported to regulate e.g. IFNγ in natural killer cells and T cells during *L. monocytogenes* or *M. bovis* BCG infection [20] or to down-regulate CASP7 during *M. avium* infection of macrophages, inhibiting apoptosis [21]. Early up-regulation of miR-29a in infected animals during our study corresponded well to down-regulation of its target CAV2. The interaction of miR-29a with its target CAV2 in humans and pigs demonstrates its conservation pointing to regulation of basal cellular signalling cascades that remained unmodified during evolution, at least in mammals. Interestingly, CAV2, CDC42 and miR-29a expression in animals that were infected with *Salmonella* but co-treated with the probiotic strain *Enterococcus faecium* NCIMB 10415 did not differ considerably. Although the molecular basis for the mode of action of probiotics remains generally unknown, one proposed mechanism is the antagonistic removal of pathogens by competition for binding sites or stimulation of epithelial barrier function [50]. Based on this idea, one could speculate that competition between probiotics and *Salmonella* impedes physical contact of the pathogens with intestinal epithelia, leading to decreased formation of FA-like structures and potentially hampering uptake. Furthermore, our microarray data indicated that *Salmonella* infection led to early down-regulation of genes involved in oxidative phosphorylation while probiotics treatment caused the opposite effect at 3 h p.i. Consistently, in a mouse model it was shown that the *Salmonella* virulence factor AvrA targets oxidative phosphorylation during early infection [51]. Since AvrA is a SPI1 T3SS effector protein, physical contact of pathogens to the host cell is needed for delivery. The mentioned model of physical competition for binding sites could explain the observed differences in expression of related genes between the groups S and SP at an early time point of infection (3 h p.i.). During established infection (3 d p.i.) involving replication and shedding of *Salmonella* this effect may be of secondary importance explaining the lack of differences between *Salmonella* and probiotics treated samples at this time point (figure 1 B).

In a recent study, Shatseva and colleagues have shown that miR-199a-3p modulates tumour cell proliferation as well as survival by regulating CAV2 [16]. Although CDC42 was predicted to be a miR-199a-3p target, CAV2 and CDC42 possessed negatively correlated expression in the studied tumour cell line MT1. Based on this observation, the authors speculated about possible transcriptional effects of CAV2 on CDC42. RAC1 and CDC42 are major Rho GTPases during cellular *Salmonella* infection and their activation through *Salmonella* effector proteins leads to formation of filopodia and uptake of pathogens [5]. Therefore, we investigated CDC42 expression in infected animals and after RNAi as well as CAV2 overexpression experiments. Our data do not support the mentioned hypothesis since intestinal *Salmonella* infection led to significantly anti-correlated expression of miR-29a and CAV2 on transcriptional as well as translational level, while an obvious linkage to CDC42 was neither detected in vivo nor in vitro. Furthermore, neither knock-down of CAV2 using specific siRNA nor overexpression of CAV2 in intestinal epithelial cells had an apparent effect on CDC42 expression proving no transcriptional or translational regulative effects of CAV2 on CDC42 in human as well as porcine intestinal cells. miR-29a was reported to target CDC42 in HeLa cells [35]. However, our RNAi experiments showed no obvious interaction between miR-29a mimic as well as antagonists and CDC42 protein levels in intestinal cells, while CAV2 was markedly down-regulated by miR-29a mimic in same cells. These observations suggest that regulation of CDC42 by miR-29a seems to be cell or tissue specific and needs to be investigated more in depth. On a different note, CAV1 deficient fibroblasts were shown to lack also CAV2 [52] and possess decreased Rho but slightly increased RAC and CDC42 activities [53]. In contrast, CAV2 deficient mice express CAV1 and are able to form caveolae, where CAV1 maintains its localisation in the plasma membrane [54]. Almeida et al. showed that LPS challenge of CAV2 deficient mice leads to STAT1 hyperactivation in intestinal cells by tyrosine 701 phosphorylation (pY701STAT1) while CAV1 deficient cells possess decreased levels of phosphorylation [55]. In Ras-transformed MEFs it was shown that site specific STAT1 phosphorylation regulates cellular protein levels of RhoA, RAC1 and CDC42. While STAT1 S727A reduced cellular levels of all three Rho GTPases, STAT1 Y701F had comparable effects with STAT1 wt [56]. Interestingly, in a recent study Schmitt and colleagues showed that IFNγ induced STAT1 phosphorylation (pY701STAT1) resulted in up-regulation of miR-29a [57]. We deduce that a feedback loop may exist consisting of miR-29a mediated CAV2 down-regulation which results in increased STAT1 phosphorylation (pY701STAT1) which in turn may positively feedback to miR-29a transcription. Since our analysis showed that CAV1 is not a miR-29a target, it appears that the signalling networks in miR-29a mediated CAV2 regulation is distinct from CAV1 and need to be investigated more in-depth in future studies.

It was highly interesting to find out that CAV2 manipulation by means of RNAi as well as overexpression directly influenced bacterial uptake in HT-29. Since our results proved that CDC42 levels in intestinal epithelial cells are influenced neither by miR-29a nor by CAV2, epithelial uptake/invasion of *Salmonella* seems not to depend on cellular concentrations of CDC42. As a result, we reasoned that CAV2 is either directly involved in *Salmonella* uptake or plays a role in signalling pathways such as regulation of the actin cytoskeleton, which is a prerequisite for pathogen invasion. As mentioned above, it was suggested that CAV2 may have a role in FAK signalling [46]. In addition, it is known that CAV1 acts as a CDC42 GDI [39] and a recent study has shown that down-regulation of RhoGDI1 in HeLa cells dramatically reduces cellular levels of CDC42 as well as RAC1 [58]. Therefore, we investigated, if CAV2 is able to modulate the activation state of CDC42 as well as its cellular levels in enterocytes. Interestingly, our RNAi and CAV2 overexpression studies showed that CAV2 is directly involved in the activation cascade of CDC42. RNAi mediated decreased levels of CAV2 in HT-29 resulted in increased cellular levels of active CDC42. By means of Co-IP experiments we were able to show that CAV2 is able to form cellular aggregates with CDC42. These observations may explain higher *Salmonella* uptake after decreased cellular CAV2, suggesting that it functions as CDC42 GDI. On the other hand and according to the study of Boulter et al. [58] one could speculate that less GDI function represented by miR-29a mediated CAV2 down-regulation would be correlated with less CDC42 in infected animals. Although, CDC42 mRNA is slightly decreased only on day 3 p.i. there was no difference in CDC42 protein levels of infected animals. This observation is supported by our in vitro data showing that cellular levels of CDC42 remained apparently unaffected in intestinal cell lines after CAV2 modulation (figure 6) suggesting different regulation mechanisms in enterocytes. Nevertheless, cellular aggregate formation between CDC42 and CAV2 as shown by the Co-IP experiments suggests that CAV2 has regulative functions and provides a basis for our future research.

Our animal experiments showed that CAV2 down-regulation coincides with increased miR-29a expression in *Salmonella* infected animals compared with controls. Furthermore, modulation of CAV2 levels in HT-29 by RNAi led to increased *Salmonella* uptake as well as CDC42 activity but apparently not RAC1. It is known that the main *Salmonella* effector protein SopE (a G-nucleotide exchange factor) potently activates both RAC1 and CDC42 causing membrane ruffles and uptake [59]. miR-29a mediated CAV2 regulation in animals significantly differed between *Salmonella* infected subjects and controls only at 3 d p.i. It is known that *Salmonella* are able to replicate in infected enterocytes and epithelial turnover and desquamation can return bacteria to the lumen leading to new infection of epithelial cells and/or colonisation of the intestine [2]. We conclude that described dynamics are the reason why significant CAV2 dysregulation is shown only at 3 d p.i., where re-infection and colonisation effects are present and all infected subjects are *Salmonella* positive [22]. These multifaceted in vivo cellular processes can only be rudimentarily repeated in vitro depending on the conditions used (cell line, infection parameters etc.). Required experimental models for infection and re-infection as well as colonisation models are hard to realise in vitro because extracellular growth of released *Salmonella* constrains the significance of such experiments. This is why we decided to study only uptake after RNAi experiments and draw conclusions based on correlating the data with in vivo results. It was shown that *Salmonella* is able to induce the formation of FA like complexes and that FAK is required for *Salmonella* uptake [60]. FAK activation leads to binding of SRC that phosphorylates CAV2 on tyrosine 19 (pY19CAV2), which does not form a hetero-oligomer with CAV1 anymore [46]. Assuming that CAV2 has synergistic or autonomous GDI function, we hypothesise that *Salmonella* induced activation of FAK leads to SRC mediated phosphorylation of CAV2, resulting in its inactivation and dissociation from the CDC42 complex. This allows binding of GTP and its activation causing actin reorganisation and inducing membrane ruffling of host cells, which leads to invasion of *Salmonella*. Concomitant down-regulation of CAV2 by miR-29a may exert synergistic effects. Our results support this hypothesis; because RNAi mediated decreased cellular levels of CAV2 implicate increased activation of CDC42 and resemble potential inactivation of CAV2 after phosphorylation.

There is a clear agreement that improved knowledge about molecular details by which bacterial pathogens modulate host cell function such as apoptosis or uptake will facilitate new therapeutic strategies [61]. Our in vitro data show that miR-29a mediated down-regulation of CAV2 in enterocytes leads both to activation of CDC42 and increased *Salmonella* uptake. Therefore, we hypothesise that determined interactions could be considered as a basis for the development of RNAi based therapeutic strategies. Inhibition of uptake or invasion would be of primary interest in the context of establishing intestinal *Salmonella* infection, where re-infection and colonisation occurs. Many drug discovery programmes have focused on development of RNAi based therapeutics. While miR-122 antagonists are shown to be successful for treatment of Hepatitis C virus infection and are currently in phase 2 clinical trials [62] there are no similar achievements regarding bacterial pathogens [63]. Our findings suggest a potentially new pathogenic mechanism of *Salmonella* promoting cellular invasion by means of miR-29a mediated CAV2 regulation. Our future work will concentrate on the corroboration of our hypothesis allowing the search for molecules or the design and specific delivery of therapeutic siRNA for interrupting the CAV2 signalling pathway. These approaches may pave the way for new therapies for humans and livestock as well as reduce use of antibiotics in the future.

### Conclusions

The genus *Salmonella* is the major cause of human foodborne illness. An approach to combat pathogens is to understand how the host organism reacts against a pathogen and to develop supportive therapeutics. Along these lines, we studied regulative networks in intestinal *Salmonella* infections of a mammal using a piglet model. We were able to outline interacting networks of miRNAs and their targets in intestinal *Salmonella* infection that mainly seem to govern focal adhesion and actin cytoskeleton. We found out that the focal adhesion protein CAV2 is involved in *Salmonella* uptake by regulating the activation state of the small Rho GTPase CDC42 in human intestinal epithelial cells. This knowledge may support the discovery of alternative therapeutic measures to impair uptake of intracellular pathogens.

## Supporting Information

Figure S1 **The figure reflects negatively correlated miRNA-mRNA interactions demonstrated as a network using Cytoscape.** This network depicts a theoretical outline of regulating miRNAs (triangles) and their potential target mRNAs (circles) while averaged anti-correlation of miRNA expression is indicated according to the colour scale below.
(TIF)

Figure S2 **Figures A and B show real-time proliferation assays using the xCELLigence RTCA system (Roche) after RNAi and overexpression of CAV2 in porcine IPEC-J2 intestinal cell line.** Graphs show means of individually treated samples while bars indicate the standard deviation of six individually measured replicates.
(TIF)

Table S1 **Determined clusters after mRNA specific microarrays including log 2 ratios and determined pathway involvement.**
(XLS)

Table S2 **Determined clusters after miRNA specific microarrays including log 2 ratios.**
(XLS)

Table S3 **Identified miRNA and mRNA targets (MAGIA analysis) possessing anti-correlated expression (correlation coefficient ≤ −0.5).**
(XLS)

Table S4 **List of oligonucleotides used in this work.**
(XLS)
