# Supplementary figures and images for: Intestinal Salmonella typhimurium Infection Leads to miR-29a Induced Caveolin 2 Regulation
Source: PLoS One. 2013 Jun 24;8(6):e67300. doi: 10.1371/journal.pone.0067300 (PMC3691122; doi:10.1371/journal.pone.0067300)

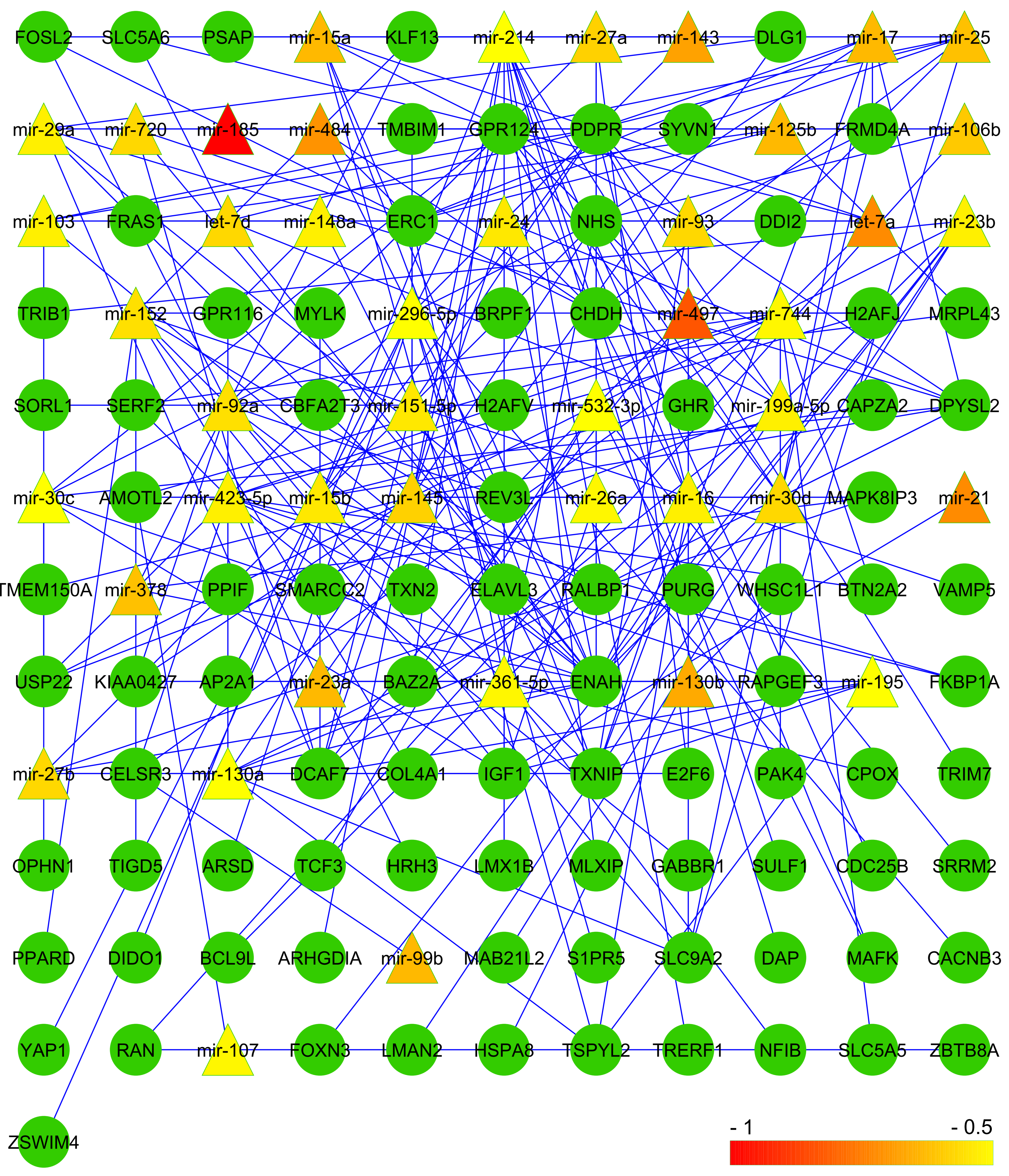

Supplement: Figure S1 — The figure reflects negatively correlated miRNA-mRNA interactions demonstrated as a network using Cytoscape. This network depicts a theoretical outline of regulating miRNAs (triangles) and their potential target mRNAs (circles) while averaged anti-correlation of miRNA expression is indicated according to the colour scale below. (TIF) [file pone.0067300.s001.tif]

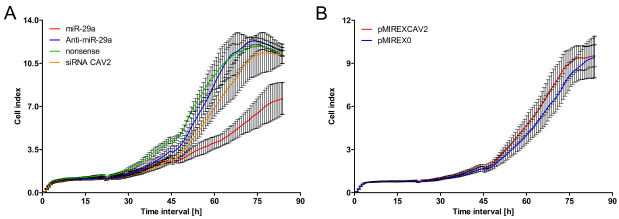

Supplement: Figure S2 — Figures A and B show real-time proliferation assays using the xCELLigence RTCA system (Roche) after RNAi and overexpression of CAV2 in porcine IPEC-J2 intestinal cell line. Graphs show means of individually treated samples while bars indicate the standard deviation of six individually measured replicates. (TIF) [file pone.0067300.s002.tif]
